# Hantavirus Infection in Children—A Pilot Study of Single Regional Center

**DOI:** 10.3390/v15040872

**Published:** 2023-03-29

**Authors:** Adriana Mocanu, Ana-Maria Cajvan, Tudor Ilie Lazaruc, Vasile Valeriu Lupu, Laura Florescu, Ancuta Lupu, Roxana Alexandra Bogos, Ileana Ioniuc, Georgiana Scurtu, Felicia Dragan, Iuliana Magdalena Starcea

**Affiliations:** 1 Pediatrics “Grigore T. Popa”, University of Medicine and Pharmacy, 700115 Iasi, Romaniaileanaioniuc@yahoo.com (I.I.);; 2 Nephrology Division, St. Mary’s Emergency Hospital for Children, 700309 Iasi, Romania; 3 Faculty of Medicine and Pharmacy, University of Oradea, 410087 Oradea, Romania

**Keywords:** acute kidney injury, children, hemodialysis, hantavirus

## Abstract

Background: Hantaviruses are infectious etiological agents of a group of rodent-borne hemorrhagic fevers, with two types of clinical manifestations in humans: hantavirus pulmonary syndrome (HPS) and hemorrhagic fever with renal syndrome (HFRS). According to available statistics, the disease occurs mainly in adults, but the lower incidence in the pediatric population might also be related to a lack of diagnosis possibilities or even unsatisfactory knowledge about the disease. Materials and Methods: The purpose of this study was to evaluate the cases of hemorrhagic fever with renal syndrome diagnosed and treated in the Department of Nephrology at St. Mary’s Emergency Hospital for Children in Iasi, Romania, representative of the North-East of Romania. We also reviewed the specialized literature on the topic. Results: Between January 2017 and January 2022, eight cases of HFRS, all men, and seven from rural areas, aged 11–18 years old, were referred to our clinic because of an acute kidney injury (AKI). Seven cases were identified as Dobrava serotype while one case was determined by Haantan serotype. Conclusions: HFRS should always be considered as a differential diagnosis when faced with a patient with AKI and thrombocytopenia. Dobrava serotype is the most common hantavirus subtype in the Balkans. For the specific prevention of human infections, mainly in high-risk groups, vaccines are needed. As far as we know, this is the first study on HFRS in Romanian children.

## 1. Introduction

Hantaviruses are the etiologic agents of a diverse group of rodent-borne hemorrhagic fevers belonging to the *Bunyavirales* order, which contains a group of single-stranded, spherical, enveloped RNA viruses. Different viral families in the *Bunyavirales* order cause hemorrhagic fever. They include *Phenuiviridae*, *Arenaviridae*, *Nairoviridae*, and *Hantaviridae* [1]. The Hantaan virus (HTNV) and related viruses have monopartite, trisegmented, negative-sense RNA genomes and produce enveloped virions [2]. After their first isolation in the 1970s, these viruses were assigned to a distinct genus, a former Hantavirus, included in the former family *Bunyaviridae*, which is the largest family of negative-sense RNA viruses that infect vertebrates, invertebrates, and plants [3]. Recent studies have led to the discovery of many similar viruses. In 2017, the former bunyavirid genus *Hantavirus* was promoted to the *Bunyavirales* order to become the current family *Hantaviridae* [4]. Hantaviruses are ubiquitous, with the exception of Antarctica [5]. In recent decades, a significant number of hitherto underestimated pathogens have emerged as fundamental threats to the health of the human population, despite the exceptional efforts of modern medicine to eradicate infectious diseases [6]. Almost all incidents caused by such pathogens are attributed to zoonotic agents that have expanded their host range and crossed species barriers. Although rare, hantavirus infections are also increasingly emerging, especially in Europe, and new serotypes with a pathogenic impact are being discovered in different parts of the world, requiring joint efforts of public health specialists, nephrologists, and infectious disease specialists [7].

The spread of the viruses is determined by the distribution of the vector and host species: viruses can be spread by arthropods (mosquitoes, ticks, sandflies) or rodents, and can cause mild to severe disease in humans or animals [8]. Many of the viruses in the order *Bunyavirales* (except the phleboviruses) cannot spread from person to person. With few exceptions, each hantavirus that is pathogenic for humans is associated with a distinct rodent species [9].

Humans become infected after contact with saliva or excreta—urine, feces—from infected rodents (or contaminated material), by inhalation of aerosols, or by direct contact of infectious materials with damaged skin or mucous membranes [10]. Hantavirus can lead to two types of infection in humans: HPS and HFRS. Hantavirus infections are recognized infrequently in the pediatric age group, with the disease occurring primarily in adults, with less than 10–15% of cases diagnosed in children [11].

Hemorrhagic fever with renal syndrome is a rodent-borne disease caused by hantaviruses, including HTNV, Seoul (SEOV), Dobrava-Belgrade virus (DOB), and Saaremma and Puumala (PUU). Most patients with HFRS are infected by direct exposure to aerosolized droppings or the body fluids of infected rodents. Human-to-human transmission is rare. The main clinical manifestations include fever, vomiting, abdominal pain, hypotension, renal damage, thrombocytopenia, and shock [12]. Epidemiologically speaking, hantavirus infections in humans are generally associated with changes in the behavior of human populations that result in large-scale exposure of persons to rodent-infested areas (military, agricultural, or forestry activities) or environmental changes that result in rapid increases of rodent host species (as in increased availability of food or preferred habitat) [2]. Although records of suspected HFRS infection can be traced back to the 1930s in Russia, Japan, and China, the disease did not capture the attention of modern society until the Korean War in 1950. From 1950 to 1953, more than 3000 United Nations soldiers contracted acquired hemorrhagic fever. Outbreaks of HFRS continued to occur in Asia and Europe in the following decades. However, it remained poorly understood until 1978 when the etiological agent of HFRS—HTNV and its reservoir—*Apodemus agrarius* were discovered by Lee et al. [13]. Professor Ho Wang Lee and his colleagues detected an antigen in the lungs and kidneys of *Apodemus agrarius* in areas where hemorrhagic fever is common. Using immunofluorescence techniques, the antigen was found to react with convalescent serum obtained from Korean Hemorrhagic Fever patients. In 1978, the team named the antigen “Korean-type antigen” and isolated it from blood samples of patients with hemorrhagic fever, successfully replicating the virus in A549 cells [14].

The first serologically confirmed report of hantavirus infection was in a four-year-old child co-infected with his mother in New Mexico in 1993 [15]. In Europe and Asia, the incidence of hantaviruses in children is sporadic. After the first report in Greece, which was associated with pediatric nephropathy in 1997, other cases have been reported. Regarding HPS, isolated cases in children and adolescents have been reported in North America, where most infections probably occurred at home, favored by socioeconomic conditions and poor hygiene. Although cases of HPS in children and adolescents are rare, reports in the literature have shown similarities in clinical manifestations compared to adult patients. Exposure of children to the risk of hantavirus infection was not addressed. Thus, while cases of hantavirus in children may occur in isolation, it is necessary to consider the possibility of simultaneous infections involving family members exposed to infected rodents. Public health professionals discuss the importance of including this zoonosis as a differential diagnosis in children with fever of unknown cause [16].

This unique virus was named HTNV after the river Hantaan (South-Korea).

To our knowledge, this is the first original article that investigates pediatric cases of hantavirus with renal determination in our country.

Cases involving children are rare. This fact is supported by extracted from the European Surveillance System, which shows that in 2018, for example, out of 1826 cases of Hantavirus infections reported in Europe, 91% (1667) accounted for people aged 25 years and older. More than that, in the 15 to 24 years age group, there was a 0.5 rate per 100,000 population, while analysis for the 0 to 14 years age group showed a rate less than 0.2 per 100,000. Notification rate peaked in patients aged 45–64 years, with 0.6 cases per 100,000 population [17].

In this study, we evaluate the pediatric cases of hemorrhagic fever with renal syndrome diagnosed and treated in the Nephrology Department of a tertiary hospital in the North-East of Romania.

## 2. Materials and Methods

### 2.1. Epidemiological Study

A retrospective single-center study was carried out over a period of 5 years (January 2017 to January 2022) on a group of patients of both sexes, aged between 0 months and 18 years, hospitalized in the Nephrology Department at St. Mary’s Emergency Hospital for Children in Iasi, Romania, regarding HFRS. This is a tertiary hospital serving the pediatric population of 7 counties. The study was conducted in accordance with the Declaration of Helsinki, and approved by the Institutional Review Board of St. Mary’s Emergency Hospital for Children in Iasi, Romania (34629/23.11.2022). In order to identify the eligible patients, we performed a free text search of our hospital’s database using the search terms “hantavirus infection” and “HFRS”, while ruling out cases of Hantavirus pulmonary syndrome. The main criterion for inclusion in the study was the definite diagnosis of hemorrhagic fever with renal syndrome beginning from the clinical aspect, and by performing serological, immunological, and pathological analysis with biopsies taken from the kidney when it was possible. Our research was based on the analysis of data from patient observation charts, hospital discharge tickets, and the pathology reports. We compiled data on gender, age, biological data, viral serotype, and renal impairment.

We have selected 79 cases of patients who presented with acute kidney injury and hematological involvement, such as thrombocytopenia/anemia. Among them, 70 were confirmed to have different types of thrombotic microangiopathy (post-diarrheic hemolytic uremic syndrome, or atypical hemolytic uremic syndrome) and one with Leptospirosis infection. After excluding other types of pathology that associated AKI and hematological involvement, we identified eight unique patients with a serology-confirmed Hantavirus infection and renal involvement. All the immunology and virology analyses necessary for the diagnosis were conducted by the Public Health Institute in Bucharest, the capital of the country.

### 2.2. Serological Diagnostic

The serological diagnosis of Hantavirus infections was performed at the Public Health Institute by enzyme-linked immunosorbent assay (ELISA) based on the detection of immunoglobulin G and M responses to the recombinant nucleocapsid proteins of five viral serotypes.

### 2.3. Kidney Biopsy

For the diagnosis of one case, we performed a kidney biopsy. We made a frozen section. The tissue staining was performed with toluidine blue and Hematoxylin Eosin on the frozen section, and the examination was in optical microscopy with the magnitude ×40 and ×200.

## 3. Results

We identified eight cases of HFRS, all of which were males, aged between 11 and 18 years (mean age 15.5 years-old), predominantly living in rural environments (7/8). All patients were referred to our clinic due to AKI, two of them having an epidemiological context—one working as a shepherd and one as a forest worker. Symptoms developed 3 to 7 days before presentation, with an average onset of 4.6 days. Sociodemographic, clinical characteristics, and biological data at onset are summarized in Table 1. All patients presented with gastrointestinal symptoms, while fever was present in 5/8 cases. Because of AKI with marked renal failure, 3 patients underwent a short course of dialysis, the rest receiving only supportive care. Viral serotype was assessed in all eight cases, revealing Dobrava serotype in seven patients and Hantan serotype in one patient. All patients survived, with complete restitution of renal function. The hospitalization period ranged between 5 to 22 days, with an average of 9.9 days. Assessment of renal function at onset and in dynamic revealed varying degrees of nitrogen retention, as seen in Table 2.

**Figure 1:**
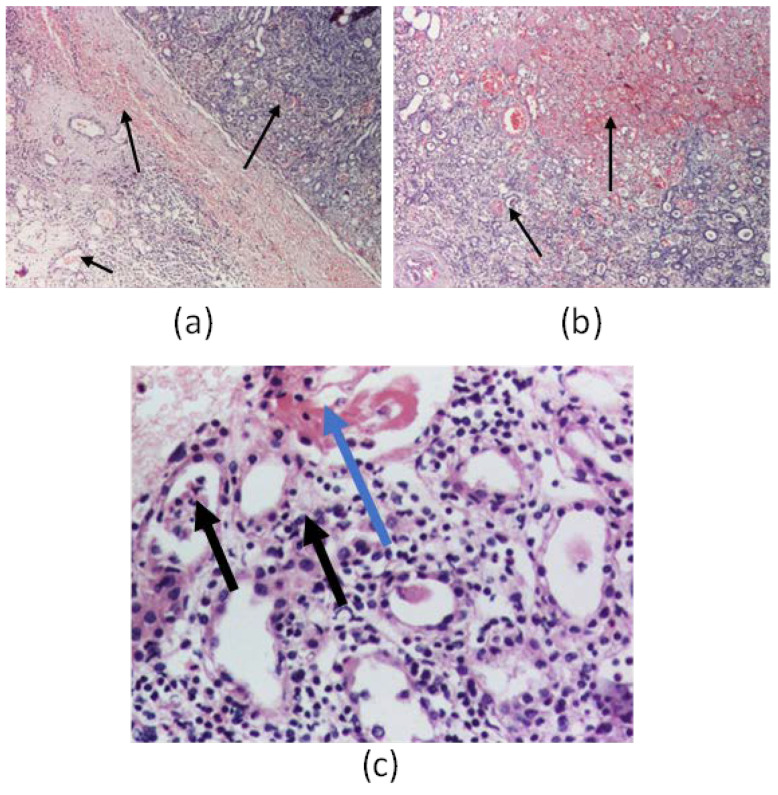
Kidney biopsy: (**a**)—Hematoxylin-Eosin (HE) coloration in optic microscopy (OM) (×40) showing multifocal interstitial hemorrhages in the subcapsular areas (black arrows); (**b**)—HE coloration in OM (×40), showing multifocal interstitial hemorrhages (black arrows); (**c**)—HE coloration in OM (×200), showing interstitial nephritis infiltrates with polymorphonuclear cells and eosinophils in the renal interstitial and tubules (black arrows); renal tubules with fibrino-leukocyte cylinders—(blue arrow).

**Figure 2:**
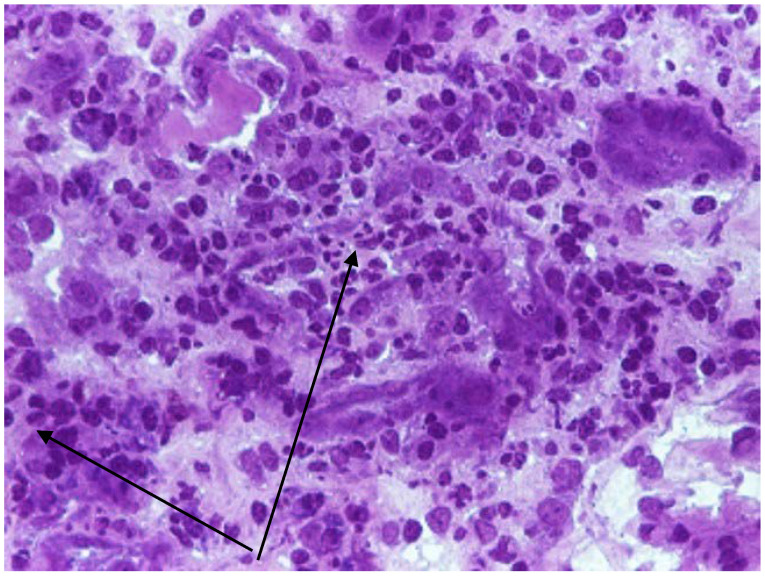
Kidney biopsy—Interstitial Nephritis. Toluidine Blue coloration in optic microscopy (×200) showing infiltrates with polymorphonuclear cells in the renal interstitium and tubules—black arrows).

On one patient we performed a renal biopsy for the differential diagnosis with a glomerulonephritis. The result showed infiltrates with polymorphonuclear cells and eosinophils in the renal interstitium and tubules, tubes with fibrino-leukocyte cylinders, suggestive of interstitial nephritis and multifocal interstitial hemorrhages (subcapsular and medullar areas), as seen in Figure 1 and Figure 2. We also performed immunofluorescence, which was negative for C1q, C3, IgA, IgM, IgG, and fibrinogen. From 22 glomeruli resulting from a needle biopsy, 18 were normal glomeruli and the rest were with moderately increased mesangial cellularity. Unfortunately, we could not perform the electron microscopy.

## 4. Discussion

### 4.1. Epidemiology of Hantavirus Infection

Based on the geographic areas in which they are found, hantaviruses are conventionally divided into two categories: Old World (Euro-Asia) hantaviruses and New World (America) hantaviruses. Amur virus (AMV), Seoul virus (SEOV), HTNV, Dobrava virus (DOBV), Tula virus (TULV), and Puumala virus (PUUV) are Old World pathogenic hantaviruses that cause HFRS in humans. This virus’s serotypes predominantly cause HFRS, a disease characterized by renal failure, hemorrhage, and shock [18]. HTN virus and DOB virus tend to produce the most severe form of the disease, with mortality rates of approximately 5.0–10.0%, while Puumala virus is endemic in northern Europe and usually causes a less severe HFRS disease, also called epidemic nephropathy (NE), with a low mortality rate of 0.1–0.2% [19]. The first New World pathogen hantavirus (Sin Nombre virus) causing HPS was discovered in the early 1990s in the Four Corners region of the USA, resulting in the second hantavirus outbreak [20]. In the same year (1990), it was discovered in Brazil, and 5 years later, in 1995, Andes virus (ANDV) was first recognized in Chile [21]. Andes hantavirus causes cardiopulmonary syndrome, being the only hantavirus for which person-to-person transmission has been reported [22,23]. It is not clear why person-to-person transmission has been documented for ANDV but not for other hantaviruses. Associated risk factors were sexual contact, deep kissing, or sleeping in the same bed. Puumala virus RNA was detected by RT-PCR but not by cell culture in the saliva from patients who had hemorrhagic fever with renal syndrome [23]. RT-PCR testing has found ANDV RNA in previous studies and in blood and other body fluids, including gingival crevicular fluid, saliva, endotracheal fluid, and urine [22].

Breast milk may contain ANDV-infected cells, leading to direct inoculation into the Peyer’s patches of the naturally fed infant [24]. It is known that the newborn has a more alkaline gastric pH, and the gastric emptying is achieved quickly in the natural diet, which favors the transmission of this viral serotype through breast milk [25].

The discovery of hantavirus outbreaks in the United States in the early 1990s fundamentally changed our knowledge of the specific clinical picture of hantavirus, mortality, origin, and route of transmission to humans. The spread of hantaviruses throughout Europe is not uniform; for example, in the 2019′s Annual Epidemiological Report from the European Centre for Disease Control, from 2015 until 2019, in Romania, there were only 23 cases of HFRS reported compared to Finland with 6627 cases or Germany with 4611 cases [26]. While for the natural reservoir, the rodents, the hantaviruses lead to a chronic infection, the human infection can clinically express itself in two forms: the hantavirus pulmonary syndrome (caused by hantaviruses of the New World—more characteristic to the American continent) and the HFRS that is caused by hantaviruses of the Old World. There are no known diseases associated with hantavirus infections in rodents, underscoring the amicable relationship between virus and host developed through mutual interaction over thousands of years. Antibodies against hantaviruses are also present in animals such as cats, dogs, pigs, cattle, and deer. Domestic animals and rodents live together in a similar habitat. Therefore, the transmission of hantaviruses from rodents to domestic animals is possible if the target organs possess suitable receptors for the virus, which allow its entry and replication. The new environment exerts an evolutionary pressure on the virus, forcing it to adapt and to probably adopt a much more pathogenic form compared to that determined in the original host [20]. HFRS is the most common type of hantavirus infection in Europe, and there are at least four serotypes that can be etiologically linked to its occurrence: the HTNV, the cause of classic HFRS, is distributed across eastern Russia; the Puumala virus, the etiologic agent of the milder HFRS variant, is the sole cause of HFRS in Nordic countries; the Dobrava virus’s known distribution is the Balkan region; and the Saarema virus in the Balkans, too. The Seoul virus is more frequent in Eastern Asia, but there were some cases reported in Western Europe (Great Britain) [11,27].

In Romania, the first case of HFRS serologically confirmed was in 2005, in a man spending a fortnight in his forest hut (C.S. Ceianu, unpublished). There aren’t many studies in our country regarding hantavirus infection in general, and almost none regarding the pediatric population. Maftei et al. published a first study in 2011 regarding the HFRS in adults from Romania since laboratory diagnosis of hantavirus infection has become available in the country, demonstrating that there are hantavirus infections in Romania and that this type of infection should not be overlooked [28]. We can, however, compare this with the studies made for the Balkan region in which our country is included, having been demonstrated that all the countries from this region have similarities regarding hantavirus infection [29]. Most frequently, the patients are male, with an overall male: female ratio of 2:1. Hantavirus diseases also occur almost exclusively in rural areas, and people at increased risk of infection are those who live or work in in specific environments linked to virus reservoirs, such as hunters, farmers, forestry workers, or military.

In our study, we have selected 78 cases of patients who presented with acute kidney injury and hematological involvement, such as thrombocytopenia/anemia. Among them, 70 were confirmed to have different types of thrombotic microangiopathy (post-diarrhea hemolytic uremic syndrome, or atypical hemolytic uremic syndrome). Eight cases were with HFRS. In our patients, all of them were male, with seven out of eight being from rural places, and only two worked in environments at risk: a forest worker and a shepherd. Regarding age, seven out of eight were 15 to 18 years of age, and one was 11. We considered that this is the range of age when children are capable of completing labor in forest environments and/or farming, and are thus more exposed to rodent-borne infections than the other age categories of childhood. The national seroprevalence of this disease is, according to the Institute of Public Health, 6.08%, a percentage similar to that found in neighboring countries among people in the risk group. According to the Institute of Public Health, cases of hantavirus infection were registered in Romania, especially in Moldova, with a seroprevalence rate of 6.08%, a percentage similar to that found in neighboring countries among people in the risk group. In Moldova, cases of hemorrhagic fever with renal syndrome have been reported since 1968 and 1977 [30]. According to the Annual Epidemiological Report for 2019 from the European Centre for Disease Prevention and Control, Romania recorded 23 cases between 2015 and 2019 [9]. In our statistics for the last 8 years, out of 79 cases selected with AKI and thrombocytopenia, 70 were diagnosed with hemolytic uremic syndrome of various etiologies, one case with Leptospira infection, and eight cases with HFRS, which indicates an incidence of approximately 10% of all cases of AKI being associated with hematologic involvement. Of course, the percentage needs to be adapted at the country level, as our hospital is only representative for the northeastern part of the country. Other diseases to be taken into consideration are meningococcemia, post-streptococcal syndromes, ricketssial diseases, and brucellosis [28]. Before confirming or ruling out a hantavirus infection, it is important to acknowledge its presence, which can be challenging in Romania where healthcare professionals may not be well-versed on this disease.

### 4.2. Pathological Features of the Disease

The HFRS is characterized by hematologic abnormalities and prominent renal involvement. Damaging the micro vascularization by the virus with resulting increased vascular permeability and vasodilatation seems to play an important role in the pathogenesis of the hantavirus infection [31]. It is assumed that vascular endothelial cells are the primary target of viral particles in a hantavirus infection. Dendritic cells, epithelial epithelial cells, and mononuclear phagocytes are invaded by the virus [32]. The infection triggers an immune response by activating cytokines and cytotoxic lymphocytes. Increased CD8+ and CD4+ T cell responses were also observed [33]. More specifically, elevated levels of IL-6 have been observed to be associated with severity for both HTNV, ANDV, and PUUV [32]. Increased levels of intercellular adhesion molecule 1 (ICAM-1) are expressed on the surface of the infected cell, resulting in endothelial cell adhesion, NK cell activation, and the release of pro-inflammatory mediators [32,33,34]. However, it is still unclear whether and to what extent the activation of NK cells may explain the pathogenesis of hantavirus infection [32]. Early in hantavirus infection, a greater increase in CD8+ T cells than CD4+ T cells was observed, and, in the acute phase of the infection, CD8+ T cells have been found to express Ki67 (marker of cell proliferation), CD38 (required for calcium signaling to trigger granule release), and HLA-DR (T cell receptor) [35]. HFRS is associated with increased production of IL-6, IL-8, IL-10, TNF, and IFN-γ [34,35]. Hantaviruses infect endothelial cells (EC), causing damage at the level of the adhesion proteins ICAM-1. Platelets and neutrophils adhere to infected EC expressing viral glycoproteins, become activated, and promote localized intravascular coagulation with the formation of thrombi and fibrin clots. At the same time, infected ECs induce the local production of bradykinin that is released into the bloodstream, which, together with the complement membrane attack complex, compromises the EC barrier function, leading to increased tissue blood flow. The typical renal histological finding is acute tubulointerstitial nephritis. Hantaviruses can infect tubular epithelial cells, glomerular endothelial cells, and podocytes of the human kidney. A high degree of proteinuria as well as medullary hemorrhages are suggestive and specific for hantavirus infection (especially for PUUV), being found in 20–60% of renal biopsies in the acute phase [36]. These lead to hypotension, hemoconcentration, thrombocytopenia, proteinuria, and leukocytosis, as well as an abrupt decline in glomerular filtration rate, with AKI [37]. In our case, the renal biopsy also showed interstitial nephritis with multifocal interstitial hemorrhages, a specific sign of infection.

### 4.3. Clinicals Features of the Disease

The hantavirus infection has to be distinguished from other acute illnesses, infectious or non-infectious. If we refer strictly to the clinical presentation, due to its diversity of signs and symptoms, there is indeed a very long list: acute abdominal pain, with vomiting and fever, can be misinterpreted as signs of acute surgical abdomen—appendicitis, inflammatory pelvic process, pancreatitis—and can lead to unnecessary surgery, acute febrile urinary infection, and bacterial sepsis [28]. We have to include the HFRS in the differential diagnosis of AKI, associated with thrombocytopenia, such as hemolytic and uremic syndrome, acute tubulointerstitial nephritis of other etiology, and acute or chronic glomerulonephritis. One of the first illnesses to rule out when facing a child with fever, thrombocytopenia, AKI, and acute hepatitis is leptospirosis, as it has a very similar clinical picture with HFRS.

The literature specifies an incubation period of 2 to 3 weeks prior, with an abrupt onset of symptoms [11]. Our patients presented themselves at the hospital at an average of 4.6 days after the onset of symptoms. The data available indicate that clinical manifestations in children are similar to and somewhat less severe than those observed in adults. Echterdiek et al. demonstrated that fever and back or loin pain represented two of the three most common clinical findings in both children and adults, but with the latter presenting significantly more often arthralgia and visual disturbances, whereas nausea/vomiting and abdominal pain were more frequently seen in children [38]. Our study emphasizes these findings, with vomiting, abdominal pain, and fever/shivers being the three most common clinical manifestations in our group.

### 4.4. Biological Features in the Disease

A hallmark of HFRS in the Balkans is renal impairment. Elevated levels of urea and creatinine are useful in early HFRS diagnosis [29]. All of our patients had various degrees of AKI, with only three out of eight needing a short course of dialysis. Echterdiek et al. found no statistical difference between children and adults concerning the need for dialysis, one of the ideas raised by their study being that pediatricians are usually more cautious in starting dialysis in children and are more accustomed to rely on clinical presentation rather than laboratory findings [38].

Thrombocytopenia is another laboratory marker for HFRS, but it is not necessary to be present, as various studies have suggested, with a low platelet count being found between 30–100% in children [26]. Generally accepted, though, is the fact that thrombocytopenia is recognized as a severity marker of hantavirus infection, with platelet count being a predictor of the severity and progression of the disease, especially marked thrombocytopenia for the subsequent severe AKI [20]. All of our patients had proteinuria and elevated C-reactive protein, consistent with the data in the literature [11].

A kidney biopsy should be considered in those children where the course of AKI does not follow the expected course towards spontaneous regression. However, particular precautions should be taken regarding the risk and benefit ratio of this invasive measure, as the majority of patients are thrombocytopenic. The most frequent type of injury described is acute tubulointerstitial nephritis, also called NE [39]. The only patient from our study who had a kidney biopsy was also afflicted with this histological aspect. In this case, the biopsy was necessary because the initial presentation needed a differential diagnosis with a glomerulonephritis. Moreover, the child did not have a suggestive history, was from an urban environment, and did not work in a forest or on a farm. In children, the evidence is scarce, but it suggests that kidney function also returns to normal, as happens in the adult population [40]. All patients from our study survived and regained complete renal function. This serves to emphasize the statement above.

### 4.5. Clinical-Evolutive Aspects in Children Hantavirosis

Until now, the clinical features and factors associated with disease severity in children with HFRS have not been well characterized. In general, the number of cases reported in children are far lower than those reported in adults. For example, Echterdiek et al. published a retrospective report in Germany regarding the clinical course of HFRS that included 317 patients from a period of almost 11 years (2006–2017), of which only 22 were children [38]. In a systematic review of the literature from 1968 to 2008, Huttunen et al. emphasized the symptoms, signs, and severity of serologically confirmed epidemic nephropathy in both pediatric and adult patients, showing that the vast majority of them were adults. From 53 papers cumulating a total number of 537 patients, only 80 were children [40]. The onset with digestive symptoms such as abdominal pain and vomiting seems to be significantly more frequent in children than in adults. Children were also more frequently hypertensive, with more severe thrombocytopenia and oliguric, compared to adults in whom polyuria, bleeding (epistaxis), and shock dominated.

In a retrospective study over a period of 9 years, conducted by Li et al. at Xi’an Children’s Hospital, several independent factors associated with disease severity were identified in the 206 children evaluated [41]. Fever, gastrointestinal symptoms, headache, back pain, bleeding of various types, and intensities as well as signs of acute kidney injury were common in children with hantavirus. Leukocytosis, decreased platelets, hematuria, proteinuria, coagulation abnormalities, increased procalcitonin, decreased glomerular filtration rate, and decreased serum Na^+^, Cl^−^, and Ca^2+^ were common laboratory findings. Hydrothorax, severe arterial hypotension requiring therapeutic support, and cerebral edema/cerebral hernia at hospital admission were independent clinical characteristics, and the percentage of neutrophils, prothrombin activity, procalcitonin, serum urea, and Ca2+ values at hospital admission were factors that independent laboratory tests associated with critical illness.

In his review, Huttunen et al. reported severe complications (especially brain haemorrhage) only in adults [40]. Described from the beginning, the association of proteinuria with hematuria is confirmed in many studies conducted on adults or children with hantavirus. An interesting finding is the correlation between the urinary excretion of interleukin-6 and the extent of proteinuria in HFRS [42]. It has also recently been reported that glucosuria on hospital admission is relatively rare, but, when present, it is a marker of AKI severity and reserved prognosis in HFRS in children and adults [43]. The conclusion made in a systematic literature review that included 53 published studies was that the evolution of HFRS seems to be less severe in children than in adults [40]. Children with hantavirus infection rarely need any invasive therapy [44]. In our small study, all patients presented with gastrointestinal symptoms, and more than half with fever. Severe AKI was proved in three patients who underwent a short course of dialysis, with the rest receiving only supportive care. The viral serotype was Dobrava serotype in seven patients and Hantaan serotype in one patient. All patients survived, with complete restitution of renal function. All these data accord with the literature.

### 4.6. Differential Diagnosis

Leptospirosis and hantavirus infection should be included in the routine differential diagnosis of acute renal failure associated with classic changes of thrombotic microangiopathy. Both leptospirosis and HFRS present with classic flu-like symptoms associated with thrombotic microangiopathy. Hemorrhagic phenomena and damage to the liver and lungs often complete the picture of the disease. In leptospirosis, cholestatic jaundice should guide the diagnosis. In addition, the differential diagnosis of hantavirus-induced HFRS should include malaria, other hemorrhagic fevers (Dengue, Yellow Fever, Influenza), non-A and non-B hepatitis, septicemia, heat shock, D+ or atypical hemolytic uremic syndrome, and thrombotic thrombocytopenic purpura [45,46]. Human immunodeficiency virus (HIV) can also infect glomerular and tubular epithelial cells, and renal expression of HIV genes plays a key role in the pathogenesis of HIV-associated nephropathy. The mechanism by which it induces podocyte damage remains unclear. Systemic vasculitis lesions may be discussed as a differential diagnosis of hantavirus.

### 4.7. Evolutive and Therapeutic Features in the Disease

Two hantaviruses, Pumala and Dobrava, are responsible for clinically manifested HFRS in patients in the Balkans [27]. From eight children included in our study, seven were infected with the Dobrava serotype. Panculescu-Gatej et al. demonstrated in a study that there are two strains of the Dobrava virus that circulated in rodent populations and transmitted to humans in Romania, and that both belong to the group of Dobrava strains circulating in Southeastern and Central Europe [47]. Till now, the literature does not specify evidence of the effectiveness of any antiviral drug for HFRS or HPS. The management of severe cases is based exclusively on supportive therapy, with correct water and electrolyte balance. HFRS patients with severe renal insufficiency may require extrarenal clearance by acute dialysis or hemodiafiltration techniques. In HCPS, patients can benefit from mechanical ventilation, and extracorporeal membrane oxygenation may even be necessary.

A double-blind, placebo-controlled clinical trial of intravenous ribavirin was conducted in China on 242 patients with HRFS, and it showed a decreased mortality seven times in cases treated early after diagnosis. At the same time, a study conducted in the European part of Russia showed a low efficacy of intravenous ribavirin treatment for HFRS caused by PUUV [29]. Most of the completed clinical trials showed that some patients developed anemia, reversible after the end of ribavirin therapy, and also hyperbilirubinemia, sinus bradycardia, and skin rashes [48]. Favipiravir, Lactoferin, and immunotherapy were tested for this hemorrhagic fever, especially for HPS, without relevant results, but with serious adverse events including dermatologic reactions, liver involvement, hypertension, and other cardiopulmonary effects [49].

Since hantavirus usually causes a self-limiting infection with a favorable evolution in 2–3 weeks, treatment is mainly supportive. Renal replacement therapy is occasionally required (in <5% of cases) and this is largely due to hypervolemia. Thus, an effective supportive treatment option is adequate, such as monitoring of fluid and electrolyte balance and the avoidance of fluid retention, especially in patients with anuria. Platelet transfusions can be provided in cases of severe thrombocytopenia with a risk of bleeding [39,50].

### 4.8. Prevention of the Disease

One of the major risk factors for hantavirus infections is the development of residential areas near forested areas. Another recognized factor is the contamination of houses or sheds with rodents. In addition, occupational exposures can affect construction or forestry workers, farmers, and soldiers [50]. People who come into contact with rodents or their droppings represent the risk group for hantavirus infection. Therefore, fighting rodents in households, in the buildings of forestry operations, and in other areas where human activities are involved is considered the most important step in preventing the disease. Ventilation of rooms, use of rubber gloves and disinfectants, and use of respirators to avoid aspiration of contaminated particles during cleaning of rodent-infested areas are all important measures that can reduce the risk of exposure to the virus.

Despite the worldwide spread of pathogenic hantaviruses and the constant efforts invested in vaccine development, there are currently no approved vaccines against these viruses [51]. Vaccinations still remain controversial: in Korea, Hantavax, a formalin-inactivated HTNV-infected mammalian mouse brain-derived vaccine, is in use but requires frequent boosters to achieve immunity for individuals at risk. Several formalin-inactivated vaccines have been used in China, but no data on their protective effect has been reported. There are no vaccines currently approved in Europe or the US. Based on recent experiences from the new SARS-CoV 2 pandemic, RNA vaccines could be a promising strategy for hantavirus infection as well [52]. The development of a vaccine is a necessary complement to the preventive actions currently applied to avoid incidents, such as the recent HPS outbreak in South America, with mortality rates above 30% [53].

Climate change influences the transmission of infectious diseases. Precipitation, humidity, and temperature were linked to human hantavirus cases in Latin America and one Caribbean country in a systematic literature review by Douglas et al. Precipitation is the climate factor most often positively associated with human hantavirus infections. Robust research on the spread of hantavirus in humans and rodents would help refine evolution prediction models [54].

## 5. Conclusions

Hantavirus infection is a rare finding in children, but HFRS must always be considered as a differential diagnosis when facing a patient with AKI and thrombocytopenia. As far as we know, this is the first study regarding HFRS in children from Romania. Although small, our cohort reflects the predominance of this infection in males, the nonspecific clinical onset with gastro-intestinal manifestations, and a self-limiting evolution of the disease, given the proper supportive treatment is assured.

The association between fever, renal failure, and thrombocytopenia prompts a serological evaluation, while their absence cannot and should not rule out this diagnosis. The Dobrava serotype is the most common subtype of the hantavirus in the Balkans, and the epidemiological context must always be searched for through a thorough medical history. The awareness regarding this rodent-borne infection should be raised both in medical personnel and for the general public, emphasizing that basic hygiene measures and reasonable precautions will greatly limit the transmission of this virus. For the specific prevention of human infections, mainly in high-risk groups, vaccines are necessary.

## Figures and Tables

**Table 1:** Sociodemographic, clinical characteristics, and biological findings at baseline.

Patients	Age, Month and Year at Admission	Baseline Characteristics	*n* = 8	Biological Findings	*n* = 8
Patient 1	*16 y.o.*	Gender		CBC	
	*November 2017*	*Female*	0	*Mild anemia*	1
Patient 2	*16 y.o.*	*Male*	8	*Leukocytosis*	2
	*October 2021*	Residence		*Thrombocytopenia*	6
Patient 3	*18 y.o.*	*Rural*	7	Inflammatory markers	
	*October 2018*	*Urban*	1	*CRP > 10 mg/dL*	8
Patient 4	*16 y.o.*	Symptoms		*ESR > 15 mm/1 h*	5
	*May 2020*	*Vomiting*	8	Hepatic cytolysis	6
Patient 5	*16 y.o.*	*Abdominal pain*	6	Urinalysis	
	*October 2019*	*Fever*	5	*No proteinuria*	0
Patient 6	*11 y.o.*	*Diarrhea*	3	*Proteinuria < 30 mg/dL*	2
	*November 2017*	*Oedema*	3	*Proteinuria 30–100 mg/dL*	2
Patient 7	*16 y.o.*	*Oligo-anuria*	2	*Proteinuria > 300 mg/dL*	4
	*January 2022*	*Headache*	2	*No hematuria*	4
Patient 8	*15 y.o.*	*Cough*	1	*Hematuria < 10 RBC/µL*	4
	*October 2022*	*Shiver*	1	*Hematuria > 10 RBC/µL*	0

CBC = complete blood count; CRP = C-Reactive Protein; ESR = Erythrocytes Sedimentation Rate; RBC = red blood cells; µL = microliters.

**Table 2:** Urea and Creatinine Values at onset versus highest value during hospitalization.

Patients	Urea (mg/dL)		Creatinine (mg/dL)	
	Onset	Highest Value	Onset	Highest Value
*Patient 1*	154	172	3.4	6.4
*Patient 2*	111	215	1.3	6.6
*Patient 3*	79	110	2.6	5.7
*Patient 4*	103	172	2.2	5.7
*Patient 5*	76	296	2.3	8.5
*Patient 6*	84	140	2.7	4.4
*Patient 7*	107	125	2.8	3.7
*Patient 8*	132	132	4	5.12

## Data Availability

The data presented in this study are available on request from the corresponding author.
